# Brucellar cervical epidural abscess - A rare cause of neck pain

**DOI:** 10.1016/j.idcr.2021.e01101

**Published:** 2021-04-01

**Authors:** Mohamed abdunasser M. Baghi, Fuad Khalil Al-Aani, Ali Rahil, Basma Ayari

**Affiliations:** Department of General Internal Medicine, Hamad General Hospital, Doha, Qatar

**Keywords:** Zoonosis infection, Brucellosis, Abscess, Epidural, Neck pain, Fever

## Abstract

Brucellosis, is a zoonosis infection with a planetary distribution, caused by facultative intracellular bacteria of the genus Brucella, thus has a variable clinical presentation. Spinal epidural abscesses are a rare presentation of the disease, and may carry risk of a neurovascular compromise. Here we report a 22-year-old male with spinal brucellosis complicated by a cervical epidural abscess. The diagnosis was based on presenting symptoms and his occupation, confirmed by laboratory investigations, blood culture and magnetic resonance imaging results. Surgical drainage of abscess was performed, followed by 6 weeks of combination antibiotic therapy. The combination of the therapeutic strategies we used lead to a significant clinical improvement in this rare case.

## Objective

Unusual clinical presentation.

## Introduction

Human brucellosis is an important public health problem in many developing countries of the Mediterranean basin, Middle East, Central Asia, and the Indian subcontinent. It is caused by a facultative intracellular a gram-negative aerobic coccobacillus bacterium of the genus Brucella and has variable clinical presentation. Common routes of infection include inoculation through cuts and abrasions in the skin or via the conjunctival sac of the eyes, inhalation of infectious aerosols, and ingestion via the gastrointestinal tract [1]. Unpasteurized dairy products are an important source of infection and represent the commonest mechanism of transmitting the disease [1,2]. Brucellosis is the most common zoonosis worldwide with approximately 500,000 cases are reported annually [2]. The incubation period is usually two to four weeks; occasionally, it may be as long as several months [3]. We hereby report a case of cervical epidural abscess caused by Brucellosis.

## Case presentation

A 22-year-old Bangladeshi gentleman with no significant past medical history, presented to emergency department with neck pain and stiffness of three days duration associated with low grade fever. There was no history of headache, photophobia, convulsion or vomiting. The patient didn’t report any history of trauma. He worked as a shepherd.

He was admitted to medical ward for further evaluation and management. On arrival, he appeared comfortable but complained of neck pain and spasm. His vitals signs on admission were; Temperature: 36.9° c, Pulse rate:74/min, Blood pressure:124/90 mmHg, Respiratory rate:21/min.

Findings on physical examination were significant for limitation in neck flexion with no neurological deficit. Other system examination were unremarkable. Initial blood investigation revealed elevation of liver enzymes with high level of C-reactive protein (CRP), mycobacterium tuberculosis PCR test was negative, Brucella serology using enzyme-linked immunosorbent assay was positive for IgG and IgM with a positive agglutination titer of 1/ 1280, blood cultures grew gram negative coccobacilli, lumbar puncture revealed normal cerebrospinal fluid cytology with negative culture Table 1. The patient was evaluated with a brain computed tomography scan (CT) which was unremarkable, regrettably the trans thoracic echocardiogram was not performed .

**Table 1:** Laboratory investigations on admission (Hospital day 1), on discharge (Hospital day 7).

Laboratory Variables	Day 1	Day 7	Reference Range
White blood cell count (x10^3^/uL)	9.8	6.3	4–10
Hemoglobin (gm/dl)	13.9	12.4	13.0–17.0
Red blood cell count (x10^6^/uL)	5.0	4.6	4.5–5.5
Hematocrit %	42.7	39.3	40–50
Platelet count (x10^3^ /uL)	204	301	150–400
Alanine transaminase (U/L)	164	130	0–41
Aspartate transaminase (U/L)	146	122	0–40
Bilirubin T (umol/L)	8	7	0–21
Alkaline phosphatase (U/L)	47	69	40–129
Creatinine (umol/L)	63	53	60–106
Urea (mmol/L)	2.7	4.2	2.8–8.1
Sodium (mmol/L)	139	135	135–145
Potassium (mmol/L)	4.1	5.5	3.5–5.1
C-reactive protein (mg/L)	51.2	20.8	0–5
Brucella Ab IgM (Titer)	Positive - 1/1280	–	–
Brucella Ab IgG (Titer)	Positive - 1/1280	–	–
Mumps virus PCR	Negative	–	–
Epstein-Barr virus PCR	Negative	–	–
Herpes simplex viruses 1–2	Negative	–	–
COV.19 PCR	Negative	–	–
TB PCR	Negative	–	–
Blood culture	Gram negative coccobacilli	–	–
Cerebral Spinal Fluid Analysis (CSF)			
CSF Appearance	Clear		–
White blood cell count (uL)	1		0–5
Red blood cell mm^3^	0		0–1
Glucose (mmol/L)	3.11		2.2–3.8
Protein (gm/L)	0.46		0.15–0.45
CSF cultures	No Growth		–

**Fig. 1:**
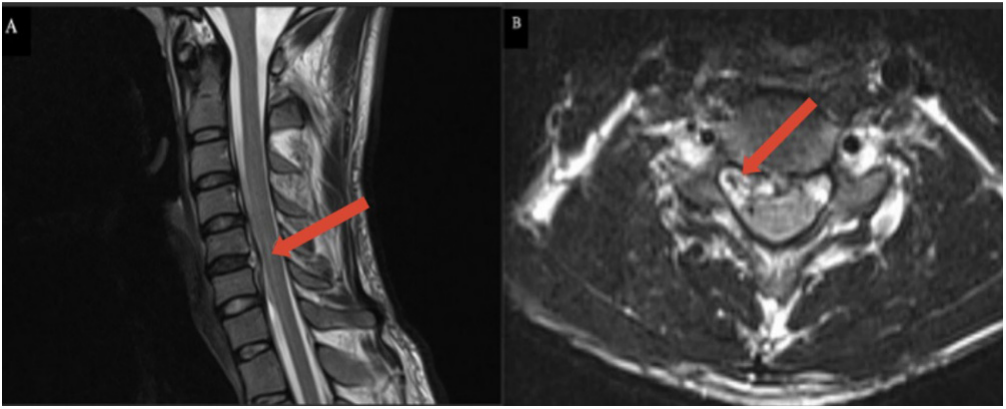
Sagittal (A) and Axial (B) views of T2-weighted image of cervical MRI showing small anterior extradural collection at C5/C6 levels extending more to the right side with mild mass effect.

Given his clinical presentation, prior history of exposure to sheeps and a positive brucella serology test, he was diagnosed as a case of acute brucellosis. The patient was started on doxycycline and aminoglycoside on the second day of admission. In view of persisting neck pain Magnetic Resonance Imaging (MRI) of cervical spine was done which showed a small anterior extradural collection at C5/C6 levels extending more to the right side with mild mass effect (Fig. 1). The patient condition was discussed with spine surgeon to evaluate the need for surgical intervention. It was decided to proceed with evacuation of the epidural collection. Exploration showed thickening of posterior longitudinal ligament with abnormal tissue attached to it. Tissue culture was taken which revel no growth, histopathological examination of tissue samples showed nonspecific inflammatory process with nonspecific granuloma.

Patient showed significant clinical improvement after the surgical evacuation with reduction of inflammatory markers (CRP decreased from 51 to 20), liver function test was not normalized but shows significant retrogression, patient discharged home in a good general condition on oral doxycycline and rifampicin to be continued for a minimum period of 6 weeks, after he receive six days in hospital gentamycin therapy.

## Discussion

Brucellosis often presents with non-specific symptoms such as fever, chills, night sweats, fatigue, myalgia, and arthralgia. Osteoarticular disease is the most common form of focal brucellosis. Sacroiliitis, spondylitis, peripheral arthritis and osteomyelitis are the most frequent bone involvement clinically encountered [4,5], with the lumbar spine being more often affected than thoracic and cervical spine. However, spinal epidural abscess is a rare presentation that carries risk for serious complications which could lead to severe long-term sequelae. Signs of brucellar epidural abscess are non-specific but can vary depending on size and location. Presentation ranges from fever, to spinal tenderness, muscular spasms or weakness, to sensory involvement and sphincter loss. Spinal tenderness is the commonest sign seen on examination. Fever is absent in half of patients [6]. In our case, the patient presented with neck pain and spasm, without fever or any focal neurological deficit. The diagnosis of brucellar spinal abscess is primarily dependent on history and clinical feature, along with MRI evidence of spinal epidural abscess and positive serum agglutination test for brucella antibody with a titer over 1:160 and/or blood culture [7]. MRI plays an important role in the diagnosis, assessment, and management of patients with spondylitis. Brucellar spondylitis may be unifocal or multifocal. Predilection for the lower lumbar spine, intact vertebral architecture despite evidence of diffuse vertebral osteomyelitis, and minimal associated para-spinal soft-tissue involvement are all features that suggest brucellar infection over other infectious diseases, including granulomatous diseases such as tuberculosis [8].

The finding of brucella organisms in blood culture is diagnostic, and culture of disk or bone tissue specimens that have been removed surgically or by needle aspiration may reveal the presence of the organism [[9], [10], [11]].

The optimal antibiotic regimen as well as duration of treatment for brucellar spinal abscess has been debated in various literatures. The World Health Organization has recommended doxycycline antibiotic therapy and rifampicin for 6 weeks [12]. Surgical treatment is indicated in case of persistence or progression of neurological deficit, spinal instability and progressive collapse of vertebrae and non-responsiveness to antimicrobial therapy [13] Percutaneous drainage or aspiration of epidural or paravertebral abscesses may be an alternative to surgery, especially for patients who are poor surgical candidates [14].

## Conclusions

As brucellosis can mimic various other clinical conditions, it is imperative to maintain a high index of suspicion particularly in high risk groups with history of direct or indirect contact with animals. The goal of medical management in brucellosis is aimed to control the symptoms and disease progression at the earliest in order to prevent complications and relapse. It is important to tailor regimen and duration of treatment according to the severity of the disease and clinical response. The cervical cord compression due to brucellar epidural abscess is rare but not uncommon and surgical intervention must be undertaken in the present of neurological deficit.

## Sources of funding

All authors have declared that they have no financial relationships at present or within the previous three years with any organizations that might have an interest in the submitted work.

## Ethical approval

Approval Taken.

## Consent

Approval was taken from IRB of medical research center of the institution.

## Author contribution

Mohamed A. Baghi: Writing, Data collection.

Fuad Al-Aani: Data collection.

Ali Rahil: Data collection.

Basma Ayari: Data collection.

## Declaration of Competing Interest

All authors declare no conflicts of interest.
